# CuO-NPs Improve Biosynthesis of Bioactive Compounds in Lettuce

**DOI:** 10.3390/plants11070912

**Published:** 2022-03-29

**Authors:** Jazmín M. Gaucin-Delgado, Adriel Ortiz-Campos, Luis G. Hernandez-Montiel, Manuel Fortis-Hernandez, Juan J. Reyes-Pérez, José A. Gonzáles-Fuentes, Pablo Preciado-Rangel

**Affiliations:** 1 Tecnológico Nacional de Mexico, Instituto Tecnológico de Torreón, Carretera Torreón-San Pedro km 7.5, Torreón 27170, Mexico; dg15860005@torreon.tecnm.mx (J.M.G.-D.); g20860018@torreon.tecnm.mx (A.O.-C.); fortismanuel@hotmail.com (M.F.-H.); 2 Centro de Investigaciones Biológicas del Noroeste, Av. Politécnico Nacional 195, Col. Playa Palo Santa Rita, La Paz 23090, Mexico; 3 Facultad de Ciencias Pecuarias, Universidad Técnica Estatal de Quevedo, Av. Quito km 1.5 vía a Santo Domingo, Quevedo 120501, Ecuador; jreyes@uteq.edu.ec; 4 Horticulture Department, Universidad Autónoma Agraria Antonio Narro, Calzada Antonio Narro 1923, Buenavista, Saltillo 25315, Mexico; jagf@uaaan.edu.mx

**Keywords:** nano-biofortification, nanoparticles, antioxidants, *Lactuca sativa* L.

## Abstract

The application of metallic nanoparticles improves the yield and content of bioactive compounds in plants. The aim of the present study was to determine the effects of the foliar application of copper nanoparticles (CuO-NPs) in the yield and content of bioactive compounds in lettuce. Different concentrations of CuO-NPs (0, 0.5, 1, 2, 4, and 6 mg mL^−1^) were applied in lettuce. The yield, nutraceutical quality, and enzymatic activity were determined. Foliar spraying of CuO-NPs induced an increase in the biosynthesis of bioactive compounds. In addition to an increase in the activity of the enzymes superoxide dismutase (SOD) and catalase (CAT) in lettuce plants, there were no negative effects on yield. Therefore, with the application of CuO-NPs, better quality lettuces are produced for the human diet due to the higher production of bioactive compounds.

## 1. Introduction

The use of nanoproducts is innovating agriculture by reducing environmental impact and increasing the yield in the plants [1]. Among the main nanoproducts are metallic nanoparticles (NPs), which can induce faster seed germination [2] and increase tolerance in the plants to biotic or abiotic stress, favoring efficient management of nutrients and plant growth [3]. In addition, it improves the yield and the content of bioactive compounds in foods of plant origin [4], which help prevent chronic and degenerative diseases, generating positive effects that allow promoting and restoring the physiological functions of the human organism [5]. In particular, the NPs based on copper (Cu) have attracted attention for agri-food purposes due to their diverse characteristics and polyvalent properties [6]. CuO-NPs have antimicrobial and antiviral properties [7], and they are also a nutritional source [8,9].

Cu is an important element in plants as it regulates various physiological and biochemical processes such as photosynthesis, respiration, carbon and nitrogen metabolism, as well as the protection against oxidative stress, among others [10]. The appropriate range of Cu in most plant species varies in low levels between 3 and 20 ppm [11], which is necessary for the development of plants [12] since most plants have a low capacity to absorb large amounts of Cu [13]. In addition, it has been reported that the copper supplied to lettuce showed tolerance to copper toxicity in concentrations of (400 μM), by altering the absorption of mineral nutrients, enzymatic activity, chlorophyll content, and leaf expansion [14]. Cu is essential for humans; however, Cu deficiency and/or excess negatively affect human health [15]. Cu deficiency leads to serious disorders such as anemia and neutropenia [10], while in excess it produces liver disorders and diseases such as Alzheimer’s, as well as nerve collapse [16,17]. Additionally, an excessive application of metallic NPs on the plants produces stress and/or toxicity, generating reactive oxygen species (ROS) and a cellular metabolism disorder. In this condition, the plants increase the content of antioxidant enzymes and non-enzymatic components for cellular protection [18], these include glutathione, vitamin C, carotenoids, ascorbate peroxidase (APX), superoxide dismutase (SOD), and catalase (CAT), among others [19].

Moreover, lettuce (*Lactuca sativa* L.) is one of the most consumed and cultivated green leafy vegetables worldwide [20]. It also has organoleptic characteristics that benefit human health and biofunctional properties related to phenolic compounds, flavonoids, vitamin C, A, E, B, potassium, magnesium, iron, calcium, and phosphorus [21]. Therefore, the objective of this study was to determine the effect of the foliar application of CuO-NPs on the accumulation of bioactive compounds in lettuce.

## 2. Results

### 2.1. CuO-NPs Effects on the Growth and Yield of Lettuce

The foliar application of CuO-NPs did not affect the yield and its components (Table 1); however, the greater number of leaves and weight per lettuce was positively affected in those treated with the dose of 6 mg mL^−1^.

### 2.2. Nutraceutical Quality

**Figure 1:**
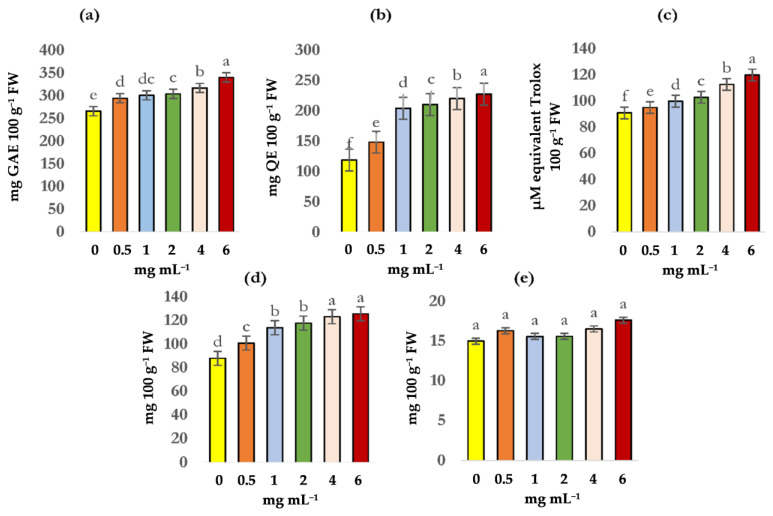
Content of total phenols (**a**), flavonoids (**b**), antioxidant capacity (**c**), chlorophyll (**d**), and vitamin C (**e**) due to the effect of different doses of CuO-NPs applied in lettuce. Data shown as mean ± standard deviation (SD). Different letters indicate a significant difference (*p* ≤ 0.05) according to Tukey’s test. GAE = gallic acid equivalents; QE = quercetin; FW = fresh weight.

The foliar spray of CuO-NPs positively modified the biosynthesis of phytochemical compounds such as the content of total phenols, flavonoids, antioxidant capacity, and chlorophyll (Figure 1a–c). The CuO-NPs positively affected the chlorophyll content in lettuce leaves (Figure 1d), inducing an increase in these pigments, the high doses increased the total chlorophyll content by 47% (Figure 1d).

### 2.3. Enzymatic Activity

**Figure 2:**
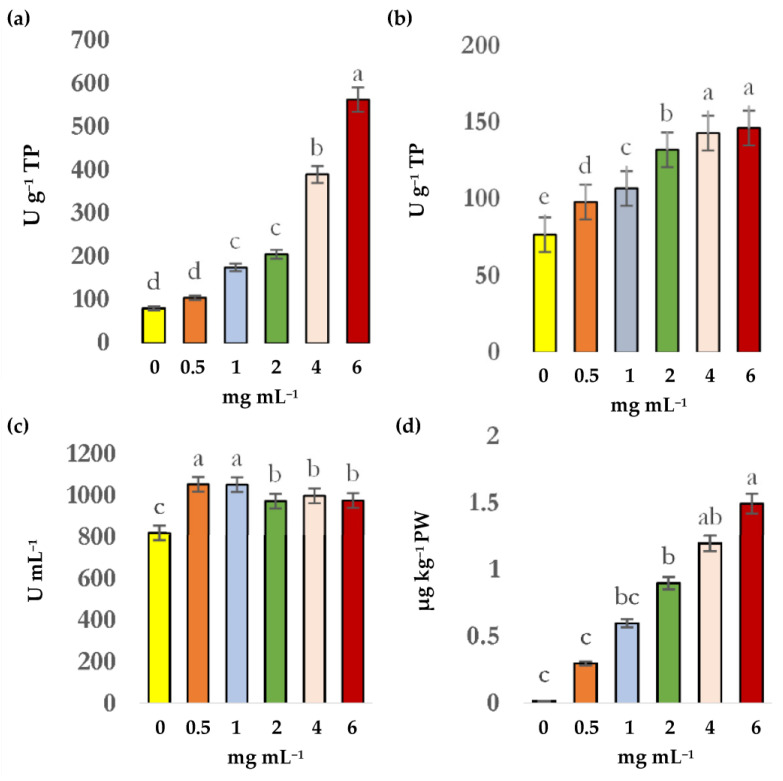
Content of glutathione peroxidase (**a**), catalase (**b**), superoxide dismutase (**c**), and copper (**d**) due to the effect of different doses of CuO-NPs applied in lettuce. Data shown as mean ± standard deviation (SD). Different letters indicate a significant difference (*p* ≤ 0.05) according to Tukey´s test. U = unit (U); TP = total proteins; PW = dry weight.

The CuO-NPs increased the enzymatic activity (Figure 2a,b) at 125% for glutathione peroxidase (GPX) and 135% for superoxide dismutase (SOD). Cu content in the leaves of lettuce increased as the sprayed doses increased (Figure 2c).

## 3. Discussion

The response of the crop to the application of CuO-NPs depends on the applied concentration [22]; since at low doses yield is promoted and high doses reduce it due to phytotoxicity [23], similar results are reported by Olkhovych et al. [24], when reporting that high doses of CuO-NPs reduce the yield of the crops, due to the fact that it increases the production of free radicals causing oxidative stress [25], and low doses improve the yield since they avoid cellular oxidation by increasing the capacity of plants to resist oxidative stress caused by reactive oxygen species under stress conditions [26]. In addition to positively affecting growth and yield, they also improve the synthesis of antioxidant compounds [27]. Another additional advantage of biosynthesized CuO-NPs is that they are less toxic than free NPs and copper sulfate [28] since bio fabricated CuO-NPs do not generate polluting by-products [29].

NPs can increase the survival of plants through many mechanisms such as improving the antioxidant defense system, increasing the absorption of water, nutrients, and phytohormones, among others [30]. Hasan et al. [31] reported that Ag-NPs increased the chlorophyll content of lettuce leaves by stabilizing the photosynthetic function; this was due to the optimal penetration into the leaf tissue. Zhao et al., [32] reported that the application of CuO-NPs increased the content of photosynthetic pigments in cucumber leaves, which was reflected in increased photosynthesis. This increase in chlorophyll content is probably due to the protection provided by metallic NPs, which may be caused by antioxidant agents in the chloroplast membrane [33]. This response depends on the dose used since CuO-NPs can induce negative responses at certain doses, while in others they can induce the opposite effect or simply have no effect [1]. This behavior, called hormesis, has been reported when metallic NPs are applied as biostimulants in cultures [34].

The beneficial effects on lettuce by CuO-NPs can be attributed to its modification in the photosynthesis system of plants with a higher transpiration rate and higher stomatal conductance [3,35]. The results indicate that CuO-NPs can be used as an agent that increases these photosynthetic compounds, which benefits the plant by participating as a coenzyme in enzyme systems involved in the formation [13] and conversion of amino acids, as well as in the detoxification of superoxide radicals. In addition, this allows them to form lignin in the cell walls [36], providing support to the vertical position in the plants, likewise, this allows them to interact in the formation of viable pollen, seed formation, and resistance to stress [37]. The vitamin C in lettuce was not affected by the concentrations of CuO-NPs used; however, the highest concentration of vitamin C corresponded to those lettuces treated with 6 mg mL^−1^ of CuO-NPs, this concentration is 53% higher than the control treatment. This study showed that CuO-NPs increased the amount of vitamin C in lettuce leaves; this could be due to the efficient stimulation of NPs in the photosynthetic apparatus by protecting it from high radiation in the light-harvesting complexes (xanthophyll cycle) [38].

Vitamin C directly influences photosynthesis because it is present in the chloroplasts, cytosol, vacuoles, and apoplastic space. In addition, it participates as an enzymatic cofactor, homeostasis of the redox system, as a precursor in the routes of synthesis of molecules of primary and secondary metabolism [39]. Likewise, green vegetables provide a greater or lesser amount of lutein and/or xanthophylls that, when in contact with vitamin C, intervene in a defense mechanism to protect photosynthesis (and therefore the chloroplast) that allows the elimination of excess energy in the form of heat safety [40]. The action of this cycle prevents the formation of O_2_ avoiding oxidative damage. It participates in the defense against biotic and abiotic oxidative stress due to its function in the degradation of H_2_O_2_ via the glutathione-ascorbate cycle [41]. López-Vargas et al. [20] indicated that applications of 250 mg L^−1^ CuO-NPs increase vitamin C, which increased the nutraceutical quality of fruits. Hernández-Hernández et al. [1] state that copper participates in the activation of enzymes, in the process of photosynthesis, the respiration of plants, and an adjuvant to these in the metabolism of carbohydrates and proteins. Vitamin C, being the most abundant antioxidant in plants, is used as a cofactor of redox enzymes due to its donation of electrons that participate in the reduction of oxidative stress in human diseases such as cancer.

Copper acts as catalytic centers for proteins in plant cell metabolism [42] and regulates the activities of enzymes such as GPX, SOD, CAT, among others, which are important scavengers of reactive oxygen species since they form the first line of defense against oxidative stress [43]. In other studies, it was shown that CuO-NPs increase CAT activity in tomato leaves [44]. Furthermore, CuO-NPs increase the activity of antioxidant enzymes such as ascorbate peroxidase (APX), superoxide dismutase (SOD), catalase (CAT), and glutathione peroxidase (GPX) [45], since these enzymes help to reduce oxidative stress [46]. On the other hand, it has been shown that the application of Cu-NPs at low concentrations (20 to 40 mg L^−1^) increase the enzymatic activity of SOD, CAT, and GPX in tomato leaves [20], rice [47], bell pepper [48] and lettuce [49]. Therefore, CuO-NPs improve the enzymatic antioxidant system of lettuce plants. In addition, CuO-NPs increase the content of PAL enzymes in plants, which act as inhibitors of the formation of singlet oxygen and free radical scavengers [50].

The use of 6.0 mg mL^−1^ favored an accumulation of this element in lettuce leaves, in direct weighting to its application. This accumulation agrees with [51], who point out that CuO-NPs increase 18.86% of the copper content in wheat grains. Similar results are also reported by Shams et al. [15], increasing the Cu content in the lettuce crop by 35%. Presumably, the constant exposure of the plant to sprays with CuO-NPs accumulated Cu in the leaves [30], thanks to the COPT1 transporter that allows the entry of Cu into the cell, which allows it to interact with the plant’s cellular metabolism [52], participating in numerous physiological processes in addition to being an essential cofactor for many metalloproteins that help in the process of photosynthesis and repair, allowing a greater translocation of this element by the plant [25]. Although the Cu concentration increased in lettuce leaves by foliar spraying of CuO-NPs, it remained safe for consumption, since between 1 and 3 mg of copper per day is required to prevent any symptoms of deficiency [53], therefore the highest dose used in the present experiment is safe to use, providing the recommended average amount in the daily intake (1200 μg day^−1^). In this sense, the use of CuO-NPs is an effective way to enrich crops, since transferring Cu to plant tissues will cause an accumulation of this micronutrient, which could help to solve Cu deficiency in the human diet [54].

## 4. Materials and Methods

### 4.1. Plant Material and Growing Conditions

The study was carried out in a greenhouse at the Instituto Tecnologico de Torreon, Coahuila, México, located at 24°30′ north latitude, 102°00′ west longitude and at 1120 m.a.s.l. Lettuce seed (*Lactuca sativa* L.) cv. Parris Island (Huertas^®^, La Huerta, México) were used, which were germinated in agricultural foam plates. Twenty days after sowing, the seedlings were transplanted into 5 kg capacity black polyethylene plastic pots containing river sand and perlite (*v*/*v*, 80:20) as growing medium previously sterilized with 5% sodium hypochlorite. The pots were distributed in double rows where a density of four plants per square meter was obtained. For crop nutrition, a Steiner nutrient solution [55] was used and applied through a drip irrigation system, providing three irrigations per day. Each plant received 0.200 L in each irrigation event, from transplantation to vegetative stage and from 1 to 1.5 L until harvest. The average temperature was 20 ± 5 °C, and the relative humidity was 40 ± 5%.

### 4.2. CuO-NPs

The CuO-NPs were donated by the Centro de Investigación en Química Aplicada (CIQA), Saltillo, Coahuila, México. The nanoparticle size was 95 nm, hemispherical morphology, dark-looking black-brown powder, with a purity of 99.8%, and they were obtained by green synthesis [56].

### 4.3. CuO-NPs Application

The evaluated treatments consisted of foliar application of CuO-NPs using the following concentrations: 0, 0.5, 1.0, 2.0, 4 and 6 mg mL^−1^ [57]. The method to prepare the different doses was using a stock solution of CuO-NPs. Subsequently, the five doses of nanoparticles were prepared in a one-liter volumetric flask, each of the nanoparticle concentrations was poured separately and completed with distilled water. The solutions were placed in manual sprayers with a capacity of 100 mL^−1^, and the different nanoparticle treatments were applied via foliar spraying during the first hours of the morning, spraying was carried out every fifteen days after transplantation (DAT).

### 4.4. Sampling

Ten plants were sampled per treatment. At 60 DAT, they were measured and weighed to determine: yield, number of leaves (NL), height leaf (HL, cm), crown diameter (CD, cm), total phenols, flavonoids, antioxidant capacity, chlorophyll, vitamin C, enzymatic activity, catalase, superoxide dismutase, and Cu content.

### 4.5. Yield

To determine the fresh weight (crop yield, g plant^−1^), the lettuce were harvested and weighed on an analytical balance (Ohaus Corporation, Pine Brook, NJ, USA).

### 4.6. Nutraceutical Quality

Preparation extract: For the determination of nutraceutical quality, 2 g of lettuce were mixed with 10 mL of ethanol at 80%. A “Stuart” shaker was used to keep the mixture stirring for 24 h. Next, the tubes were centrifuged at 120× *g* for 24 h. The supernatant (ethanolic extract, EE) was removed for analytical testing.

Total phenolic content was quantified by the Folin–Ciocalteau method [58] 50 µL of EE were used and the results were expressed as mg GAE 100 g^−1^ FW. Total antioxidant capacity was measured by the DPPH method [59], 50 µL of the EE were utilized and the results were expressed in µM equivalent Trolox 100 g^−1^ FW. Total flavonoids were quantified in a UV-Vis [1] spectrophotometer (CGOLDEN-WALL, Seattle, WA, USA) at 510 nm and expressed as mg QE 100 g^−1^ FW.

### 4.7. Photosynthetic Pigments

Chlorophyll content (mg 100 g^−1^ FW) was determined by the method of Nagata and Yamashita [60], using the equations:

Chla = 0.999 × Abs 663 − 0.0989 × Abs 645

Chlb = −0.328 × Abs 663 + 1.77 × Abs 645

### 4.8. Vitamin C

It was obtained by means of the titration method [61]. Fresh fruit samples of 10 g were used, and vitamin C content was calculated using the equation: Vic C (mg 100 g PF) = (mL of 2.6 dichlorophenolindophenol) (0.088) (total volume) (100)/(aliquot volume) (sample weight).

### 4.9. Enzymatic Activity

Samples of 200 μL of EE were used for the determination of glutathione peroxidase (GPX) by the method of Flohé and Günzler [62]. The results were expressed as a unit (U) per gram of total proteins (U TP^−1^). Samples or 100 μL of EE were utilized for the quantification of catalase (CAT) by the method of Dhindsa et al. [63] and expressed as U TP^−1^. Superoxide dismutase (SOD) was determined by SOD Cayman 706002^®^ kit. The results were expressed as U mL^−1^, where, U is defined as the amount of enzyme needed to exhibit 50% dismutation of the superoxide radical.

### 4.10. Cu Content in Lettuce Leaves

The copper concentration in lettuce leaves was determined according to the AOAC [64] by atomic absorption spectrophotometry, with an air-acetylene flame (Varian-Spectr AA 3110, Palo Alto, CA, USA), and the results were expressed in µg kg^−1^ dry weight (DW).

### 4.11. Statistical Analysis

The experimental design was completely randomized. The normality and homogeneity of the variances of the data obtained were verified using the Kolmogorov-Smirnov and Bartlett tests, respectively. Subsequently, the analysis of variance of simple classification and post hoc multiple comparison of means was performed through the Tukey HSD test at a probability of 5%, with the software SAS v 9.0 (SAS Institute, 2004, Cary, NC, USA).

## 5. Conclusions

The foliar application of CuO-NPs improves the nutraceutical quality, antioxidant capacity, and Cu concentration in lettuce leaves, in addition to increasing the activity of the enzymes (GPX, CAT, and SOD), without effects on the yield. Therefore, with the application of Cu-NPs, better quality lettuces are produced for the human diet due to the accumulation of bioactive compounds.

## Figures and Tables

**Table 1:** Yield components due to the effect of the different concentrations of CuO-NPs applied via foliar.

CuO-NPs mg mL^−1^	Yield g Plant^−1^	NL	HL	CD
			cm	
0	137.93 ± 23.63 a *	44.33 ± 7.37 a *	24.4 a ± 3.92 *	33.5 ± 1.77 a *
0.5	162.58 ± 21.58 a	51.66 ± 8.08 a	19.8 ± 4.27 a	35.8 ± 1.61 a
1	172.97 ± 19.63 a	41.67 ± 8.03 a	32.8 ± 4.29 a	35.1 ±1.79 a
2	154.47 ± 17.56 a	43.33 ± 9.05 a	23.1 ± 4.23 a	34.4 ± 1.81 a
4	192.73 ± 22.20 a	31.00 ± 9.02 a	25.7 ± 4.08 a	39.1 ± 1.94 a
6	200.73 ± 21.03 a	53.66 ± 9.32 a	25.1 ± 3.97 a	36.3 ± 1.94 a

* Data are shown as the mean ± standard deviation (SD, *n* = 30). Different letters indicate a significant difference (*p* ≤ 0.05) according to Tukey’s test. NL = number of leaves; HL = height leaf; CD = crown diameter.

## Data Availability

The data presented in this study are available in article.
